# Association of 25(OH)D status with calcium metabolism, inflammation, and thyroid autoimmunity in patients with type 2 diabetes mellitus

**DOI:** 10.3389/fendo.2026.1867755

**Published:** 2026-06-04

**Authors:** Nannan Lv, Jianjian Xiang, Jinsong Kuang, Fei Liu, Lan Cheng, Shanyu Yin

**Affiliations:** 1 Department of Endocrinology and Metabolism, The Fourth People’s Hospital of Shenyang, China Medical University, Shenyang, Liaoning, China; 2 Department of Ultrasound Medicine, The First Affiliated Hospital, Zhejiang University School of Medicine, Hangzhou, Zhejiang, China

**Keywords:** 25(OH)D deficiency, calcium metabolism, inflammation, thyroid autoimmunity, type 2 diabetes mellitus

## Abstract

**Introduction:**

25-hydroxyvitamin D [25(OH)D] deficiency is highly prevalent in type 2 diabetes mellitus (T2DM) and may contribute to immune and metabolic dysfunction. This study aimed to investigate the clinical associations of serum 25(OH)D levels with calcium metabolism, inflammation, thyroid function, and autoimmunity in patients with T2DM.

**Methods:**

This cross-sectional study enrolled 503 patients with T2DM, stratified into four groups based on 25(OH)D levels: severe deficiency (<10 ng/mL), moderate deficiency (10–20 ng/mL), insufficiency (20–30 ng/mL), and sufficiency (>30 ng/mL). Clinical and biochemical parameters were compared across groups and between thyroid autoantibody (TPOAb/TGAb) positive and negative subgroups. A multiple linear regression model identified independent predictors of 25(OH)D levels.

**Results:**

25(OH)D deficiency (<20 ng/mL) was present in 83.7% of patients. Higher 25(OH)D levels correlated with lower PTH (P = 0.010) and higher calcium (P<0.001). hsCRP peaked in the moderate deficiency group (2.66 mg/L, P<0.001). T3 and fT3 peaked in the insufficiency group and were lowest in severe deficiency (P<0.05). TGAb positivity was associated with higher 25(OH)D levels (13.45 vs. 12.00 ng/mL, P = 0.008), whereas TPOAb positivity was associated with lower levels (11.90 vs. 12.85 ng/mL, P<0.001). No significant associations were found with HbA1c, BMI, TSH, or bone markers.

**Conclusion:**

In patients with T2DM, 25(OH)D deficiency is highly prevalent and independently associated with altered calcium-PTH homeostasis, elevated inflammation, and suppressed T3 levels. The opposing relationships with TGAb and TPOAb suggest complex interactions between vitamin D and thyroid autoimmunity in this population.

## 1 Introduction

Type 2 diabetes mellitus (T2DM) and Hashimoto’s thyroiditis (HT) represent two of the most prevalent endocrine disorders worldwide, and their clinical coexistence is increasingly recognized as a significant public health concern. Epidemiological evidence confirms a bidirectional relationship: individuals with T2DM exhibit a significantly higher incidence of HT compared to the general population, while those with autoimmune thyroid disease face an elevated risk of developing impaired glucose metabolism and overt T2DM (1, 2). This frequent overlap suggests shared underlying pathophysiological mechanisms. Central to this intersection are chronic low-grade inflammation and insulin resistance, which not only characterize T2DM but also create a permissive environment for the breakdown of immune tolerance, thereby promoting autoimmune responses against thyroid tissue (3). The co-occurrence of these conditions often results in a compounded metabolic burden, complicating disease management and potentially accelerating the progression of both microvascular and macrovascular complications (4–6).

Beyond its classical role in calcium and bone homeostasis, vitamin D has emerged as a pivotal immunomodulator and metabolic regulator with pleiotropic effects. Through binding to the vitamin D receptor (25(OH)DR), which is ubiquitously expressed on pancreatic β-cells, immune cells (including T and B lymphocytes), and thyroid follicular cells, vitamin D exerts direct influence on insulin secretion, peripheral insulin sensitivity, and the differentiation of T-helper cells toward a tolerogenic phenotype (7–9). In the context of T2DM, hypovitaminosis D is robustly associated with exacerbated insulin resistance and accelerated β-cell dysfunction (10, 11). Concurrently, in HT, vitamin D deficiency correlates with elevated titers of thyroid autoantibodies (TPOAb and TGAb) and a heightened pro-inflammatory cytokine profile, suggesting its role in perpetuating autoimmune activity (12). Notably, patients with the dual diagnosis of T2DM and HT frequently present with profoundly lower serum 25-hydroxyvitamin D [25(OH)D] levels, which have been shown to inversely correlate with the duration and severity of both diseases (13, 14). This positions 25(OH)D status as a potential common soil or shared risk factor linking metabolic dysregulation and autoimmunity.

However, three key gaps remain: (1) whether vitamin D status shows non-linear associations with thyroid hormone metabolism; (2) whether TPOAb and TGAb exhibit distinct relationships with 25(OH)D; and (3) the lack of an integrated evaluation of calcium–PTH and inflammation in patients with T2DM. This study addresses these gaps by examining a comprehensive panel of clinical, metabolic, and immunological parameters in a well-defined cohort.

## 2 Materials and methods

### 2.1 Patient selection criteria and study design

This cross-sectional study was conducted at the Fourth Peoples’ Hospital of Shenyang, China Medical University, Shenyang, China, between January 2022 and December 2022, and included 1295 patients with T2DM. The inclusion criteria for this study were as follows: a confirmed diagnosis of type 2 diabetes mellitus (T2DM), age ≥18 years, the actual age range in this cohort was from 20 to 88 years. The study included both newly diagnosed (duration <1 year) and long-standing T2DM patients (duration ≥1 year). Diabetes duration was recorded for all participants. Patients were excluded if they had abnormal liver or kidney function, acute diabetic complications, a documented history of cerebrovascular or cardiovascular events within the preceding six months, malignancy, acute infection, liver disease, or pregnancy at the time of enrollment, to reduce confounding and improve internal validity.

Type 2 diabetes mellitus was diagnosed according to the 2014 American Diabetes Association (ADA) diagnostic criteria. A diagnosis was established if any of the following conditions were met: fasting plasma glucose ≥7.0 mmol/L, glycated hemoglobin (HbA1c) ≥6.5%, or 2-hour plasma glucose ≥11.1 mmol/L after a 75 g oral glucose tolerance test (OGTT) (15).

A total of 503 patients with confirmed T2DM were enrolled. According to serum 25(OH)D concentrations, participants were categorized into four groups according to internationally accepted criteria: <10 ng/mL, 10–20 ng/mL, 20–30 ng/mL, and ≥30 ng/mL. Specifically, serum 25(OH)D concentrations <10 ng/mL were classified as severe 25(OH)D deficiency, 10–20 ng/mL as 25(OH)D deficiency, 20–30 ng/mL as 25(OH)D insufficiency, and ≥30 ng/mL as 25(OH)D sufficiency (16, 17). This stratification facilitated the comparison of clinical parameters across different vitamin D status levels. Serum 25(OH)D levels were quantified using electrochemiluminescence (ROCHE cobas e801). As the stable storage form with a long half-life (2–3 weeks), its concentration accurately reflects total body reserves and is considered the clinical gold standard for 25(OH)D status assessment.

### 2.2 Data collection

Blood samples were collected between 6:00 and 8:00 a.m. after at least 8 hours of overnight fasting. All study-related tests were conducted within the hospital and validated by accredited reference laboratories to ensure the accuracy and reliability of the results.

During the physical examination, participants wore lightweight clothing and had their height measured without shoes. Body mass index (BMI) was calculated as weight in kilograms divided by the square of height in meters (kg/m^2^). The following baseline data were collected: sex, age, diabetic duration, medical history (Participants with a history of vitamin D supplementation, thyroid medication use, or corticosteroid use were excluded from the study to minimize potential confounding effects of these drugs on the studied associations), medication use and BMI. C-reactive protein (CRP) was determined by immunoturbidimetric assay (Mindray BC-7500). HbA_1_C was measured by high-performance liquid chromatography (HuiZhong MQ-6000). PTH and osteocalcin (OC) were quantified by electrochemiluminescence immunoassay (Roche cobas e801). Serum calcium and phosphorus were measured by colorimetric assays (Roche cobas c701). Thyroid hormones, TSH, TPOAb, and TGAb were measured by direct chemiluminescence immunoassay (Siemens Advia Centaur XP).

### 2.3 Statistical analysis

In this study, statistical results were expressed as the mean ± standard deviation (mean ± SD) or as the median with interquartile range [M (P25, P75)], depending on data distribution. For comparisons between groups, non-parametric statistical tests were performed, including the Mann–Whitney U test for two-group comparisons and the Kruskal-Wallis H test for comparisons among multiple groups. All tests were two-tailed, and a p-value < 0.05 was considered statistically significant.

Based on preliminary statistical analyses, clinically relevant laboratory parameters with statistical significance were included in a multivariate linear regression model. To identify independent predictors of vitamin D status (consistent with our objective to characterize determinants of vitamin D deficiency, not to test causality), Serum 25(OH)D concentration was used as the dependent variable, while TPOAb, TGAb, T3, fT3, PTH, hsCRP, and calcium (Ca) levels were entered as independent variables to construct the model. This model aimed to investigate the independent associations between these variables and serum 25(OH)D levels. Following model construction, a residual plot was generated to evaluate the model’s goodness-of-fit and to examine the distributional properties of residuals, in order to assess its suitability and robustness.

Statistical analyses were performed using SPSS (version 26.0; SPSS Inc., Chicago, IL, USA), R (version 4.5.1; R Foundation for Statistical Computing, Vienna, Austria), and MATLAB (version R2021b; MathWorks, Natick, MA, USA).

## 3 Results

### 3.1 Clinical characteristics of patients stratified by 25(OH)D levels

**Figure 1:**
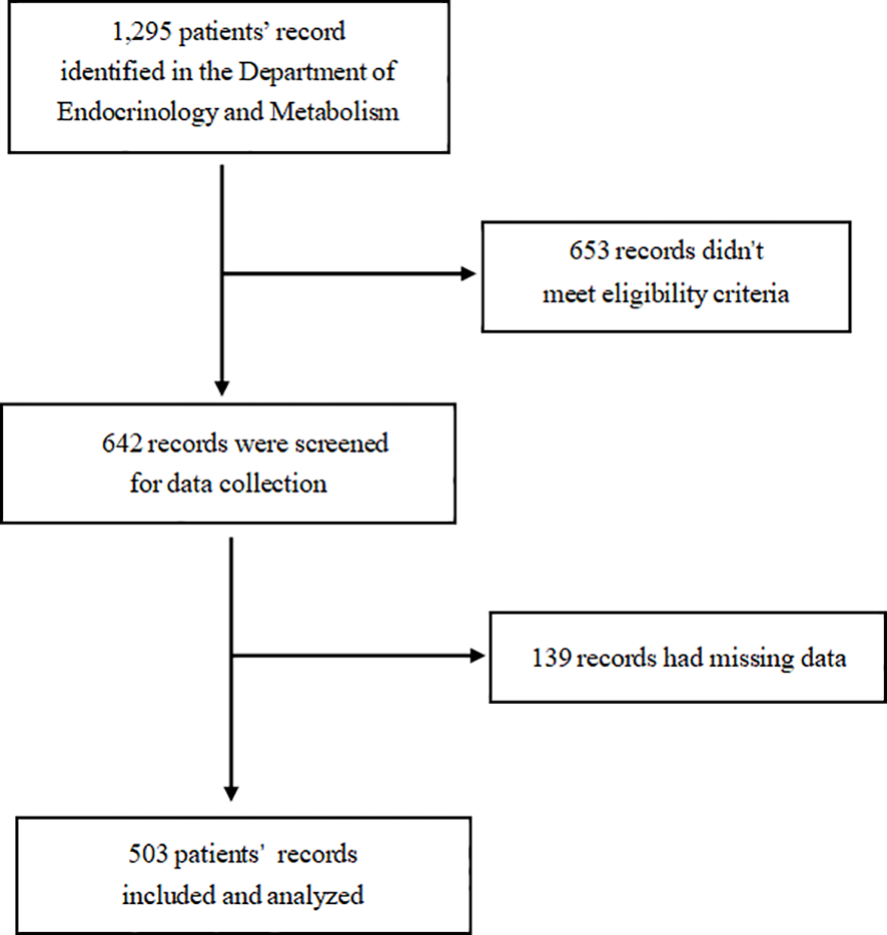
STROBE flow diagram for cross-sectional study.

A total of 503 patients with type 2 diabetes mellitus (T2DM) who met the inclusion and exclusion criteria were enrolled in this study (**Figure 1**). Baseline characteristics, stratified by serum 25(OH)D levels, are summarized in **Table 1**. The majority of participants (83.7%) exhibited 25(OH)D deficiency (<20 ng/mL), with 151, 270, 71, and 11 patients in the 0-10, 10-20, 20-30, and ≥30 ng/mL categories, respectively.

**Table 1:** Detailed characteristics of the patients according to 25(OH)D (ng/mL) levels.

Clinical laboratory indicators	Grouping criteria				P value
	0-10, (n=151)	10-20, (n=270)	20-30, (n=71)	>30, (n=11)	
Age(mean(SD)), y	57.74(9.69)	59.38(9.84)	58.99(8.78)	61.36(7.07)	0.322
Diabetic duration(mean(SD)), months	126.33(96.10)	141.53(95.81)	134.80(90.21)	146.00(93.25)	0.055
BMI(mean(SD)), kg/m^2^	24.95(3.63)	24.87(3.16)	25.11(2.79)	23.93(2.18)	0.615
T3(mean(SD)), nmol/L	1.50(0.30)	1.57(0.34)	1.75(1.16)	1.47(0.38)	0.015*
T4(mean(SD)), nmol/L	104.73(20.91)	108.76(22.91)	109.90(21.74)	115.42(30.18)	0.236
fT3(mean(SD)), U/mL	4.43(0.58)	4.65(0.90)	5.02(2.98)	4.45(0.78)	0.013*
fT4(mean(SD)), U/mL	15.54(2.42)	16.33(7.26)	15.69(2.07)	15.70(0.92)	0.811
sTSH(mean(SD)), μIU/ml	2.89(9.13)	2.23(4.27)	2.29(2.61)	1.89(1.46)	0.674
hsCRP(mean(SD)), mg/L	1.59(4.30)	2.66(6.10)	1.83(2.50)	1.82(2.00)	<0.001*
PTH(mean(SD)), pg/ml	48.56(23.86)	45.57(21.81)	42.06(19.30)	29.69(10.51)	0.010*
OC(mean(SD)), ng/ml	10.79(4.20)	10.45(4.96)	10.19(4.05)	9.90(5.12)	0.445
HbA1c(mean(SD)), %	9.20(2.21)	9.05(2.18)	9.07(1.96)	8.64(2.23)	0.442
Ca(mean(SD)), mmol/l	2.20(0.13)	2.24(0.13)	2.28(0.11)	2.33(0.09)	<0.001*
P(mean(SD)), mmol/l	1.16(0.19)	1.15(0.18)	1.12(0.14)	1.11(0.17)	0.721

The Kruskal-Wallis test was used for analysis. *indicates a statistically significant difference (P < 0.05).

Significant differences across 25(OH)D categories were observed for PTH, serum calcium, hsCRP, T3, and fT3 (Kruskal-Wallis test, all P < 0.05). Median PTH levels progressively declined with increasing 25(OH)D concentrations (48.6-29.7 pg/mL), whereas serum calcium rose across categories (2.20-2.33 mmol/L), consistent with physiological calcium-PTH feedback. In addition, hsCRP exhibited a nonlinear trend, reaching the highest median concentration (2.66 mg/L) in the 10–20 ng/mL group, suggesting a potential inflammatory threshold within the moderate deficiency range. T3 and fT3 also showed non-monotonic patterns, peaking in the 20–30 ng/mL category (T3: 1.75 nmol/L; fT3: 5.02 pg/mL) and declining in the ≥30 ng/mL group.

No significant differences were detected in age, diabetic duration, BMI, HbA1c, total T4, fT4, TSH, osteocalcin, or serum phosphorus levels (all P > 0.05).

### 3.2 Differences in clinical indicators under 25(OH)D threshold statuses

A comparative analysis across different 25(OH)D threshold groups (**Table 2**), conducted using the Mann–Whitney U test, focused on the calcium–PTH metabolic axis (PTH, Ca, P), inflammatory marker hsCRP, and thyroid function markers (T3, fT3). The results demonstrated that T3 and fT3 levels were significantly reduced exclusively in patients with severe 25(OH)D deficiency (25(OH)D <10 ng/mL, P = 0.013 and 0.003, respectively). This finding is consistent with **Table 1**, where T3 levels reached a peak in the 20–30 ng/mL group, suggesting that severe 25(OH)D deficiency may impair T3 synthesis.

**Table 2:** Differences in clinical indicators under 25(OH)D (ng/mL) threshold statuses.

Clinical laboratory indicators	Grouping criteria	P value
Age, y	25(OH)D<30, 25(OH)D>30	0.459
	25(OH)D<20, 25(OH)D>20	0.851
	25(OH)D<10, 25(OH)D>10	0.090
BMI, kg/m^2^	25(OH)D<30, 25(OH)D>30	0.298
	25(OH)D<20, 25(OH)D>20	0.657
	25(OH)D<10, 25(OH)D>10	0.911
T3, nmol/L	25(OH)D<30, 25(OH)D>30	0.247
	25(OH)D<20, 25(OH)D>20	0.099
	25(OH)D<10, 25(OH)D>10	0.013*
T4, nmol/L	25(OH)D<30, 25(OH)D>30	0.296
	25(OH)D<20, 25(OH)D>20	0.209
	25(OH)D<10, 25(OH)D>10	0.072
fT3, U/mL	25(OH)D<30, 25(OH)D>30	0.700
	25(OH)D<20, 25(OH)D>20	0.083
	25(OH)D<10, 25(OH)D>10	0.003*
fT4, U/mL	25(OH)D<30, 25(OH)D>30	0.927
	25(OH)D<20, 25(OH)D>20	0.859
	25(OH)D<10, 25(OH)D>10	0.391
sTSH, μIU/ml	25(OH)D<30, 25(OH)D>30	0.728
	25(OH)D<20, 25(OH)D>20	0.819
	25(OH)D<10, 25(OH)D>10	0.310
hsCRP, mg/L	25(OH)D<30, 25(OH)D>30	0.482
	25(OH)D<20, 25(OH)D>20	0.055
	25(OH)D<10, 25(OH)D>10	<0.001*
PTH, pg/ml	25(OH)D<30, 25(OH)D>30	0.006*
	25(OH)D<20, 25(OH)D>20	0.009*
	25(OH)D<10, 25(OH)D>10	0.118
OC, ng/ml	25(OH)D<30, 25(OH)D>30	0.438
	25(OH)D<20, 25(OH)D>20	0.351
	25(OH)D<10, 25(OH)D>10	0.142
HbA1c, %	25(OH)D<30, 25(OH)D>30	0.209
	25(OH)D<20, 25(OH)D>20	0.842
	25(OH)D<10, 25(OH)D>10	0.299
Ca, mmol/l	25(OH)D<30, 25(OH)D>30	0.003*
	25(OH)D<20, 25(OH)D>20	<0.001*
	25(OH)D<10, 25(OH)D>10	<0.001*
P, mmol/l	25(OH)D<30, 25(OH)D>30	0.372
	25(OH)D<20, 25(OH)D>20	0.088
	25(OH)D<10, 25(OH)D>10	<0.001*

The Mann-Whitney U test was used for analysis. * indicates a statistically significant difference (P < 0.05).

Significant differences were also observed for PTH and Ca in most threshold comparisons (PTH: P = 0.006, 0.009, 0.118; Ca: P = 0.003, <0.001, <0.001), indicating a robust association between 25(OH)D deficiency and the calcium-PTH metabolic axis. In addition, hsCRP levels were significantly elevated in the severe 25(OH)D deficiency group (<10 ng/mL, P < 0.001), suggesting augmented systemic inflammation.

No statistically significant differences were observed across all 25(OH)D threshold groups for metabolic indices (BMI, HbA1c), thyroid hormones (T4, fT4), TSH, bone formation marker osteocalcin (OC), or demographic variable age, although age displayed a marginal trend between 25(OH)D <10 and ≥10 ng/mL (P = 0.09).

Collectively, these findings indicate that 25(OH)D deficiency predominantly influences the calcium-PTH axis and systemic inflammation, with severe 25(OH)D deficiency associated with reduced T3 and fT3 levels, while it appears unrelated to glucose metabolism, body weight, overall thyroid hormone synthesis (T4, fT4, TSH), or bone formation (OC).

### 3.3 The association between 25(OH)D levels and thyroid autoantibodies

Based on **Tables 3**, **4**, the relationship between 25(OH)D levels and thyroid antibody status demonstrates statistically significant associations in specific subgroups. In **Table 3**, patients with positive TGAb exhibited significantly higher 25(OH)D levels than TGAb-negative patients (13.45 vs. 12.00 ng/mL, P = 0.008). Conversely, TPOAb-positive patients demonstrated lower 25(OH)D levels than TPOAb-negative patients (11.90 vs. 12.85 ng/mL, P < 0.001).

**Table 3:** Differences in 25(OH)D levels according to antibody groups.

Antibody		25(OH)D (ng/mL)	n	P value
TGAb, U/mL	Negative	12.00 (9.18–15.93)	269	0.008*
	Positive	13.45 (9.80–19.20)	234	
TPOAb, U/mL	Negative	12.85 (9.50–17.90)	424	<0.001*
	Positive	11.90 (8.60–15.80)	79	

The Mann-Whitney U test was used for analysis. *indicates a statistically significant difference (P < 0.05).

**Table 4:** Test results of antibody positivity rates according to 25(OH)D (ng/mL) levels (0–10, 10–20, 20–30, >30).

Antibody		0–10	10–20	20–30	>30	P value
TGAb	Negative	91	145	29	4	0.033*
	Positive	60	125	42	7	
TPOAb	Negative	120	234	60	10	0.258
	Positive	31	36	11	1	
TGAb & TPOAb	Negative	82	136	26	4	0.618
	Positive	22	27	8	1	

The Chi-square test was used for analysis. *indicates a statistically significant difference (P < 0.05).

**Figure 2:**
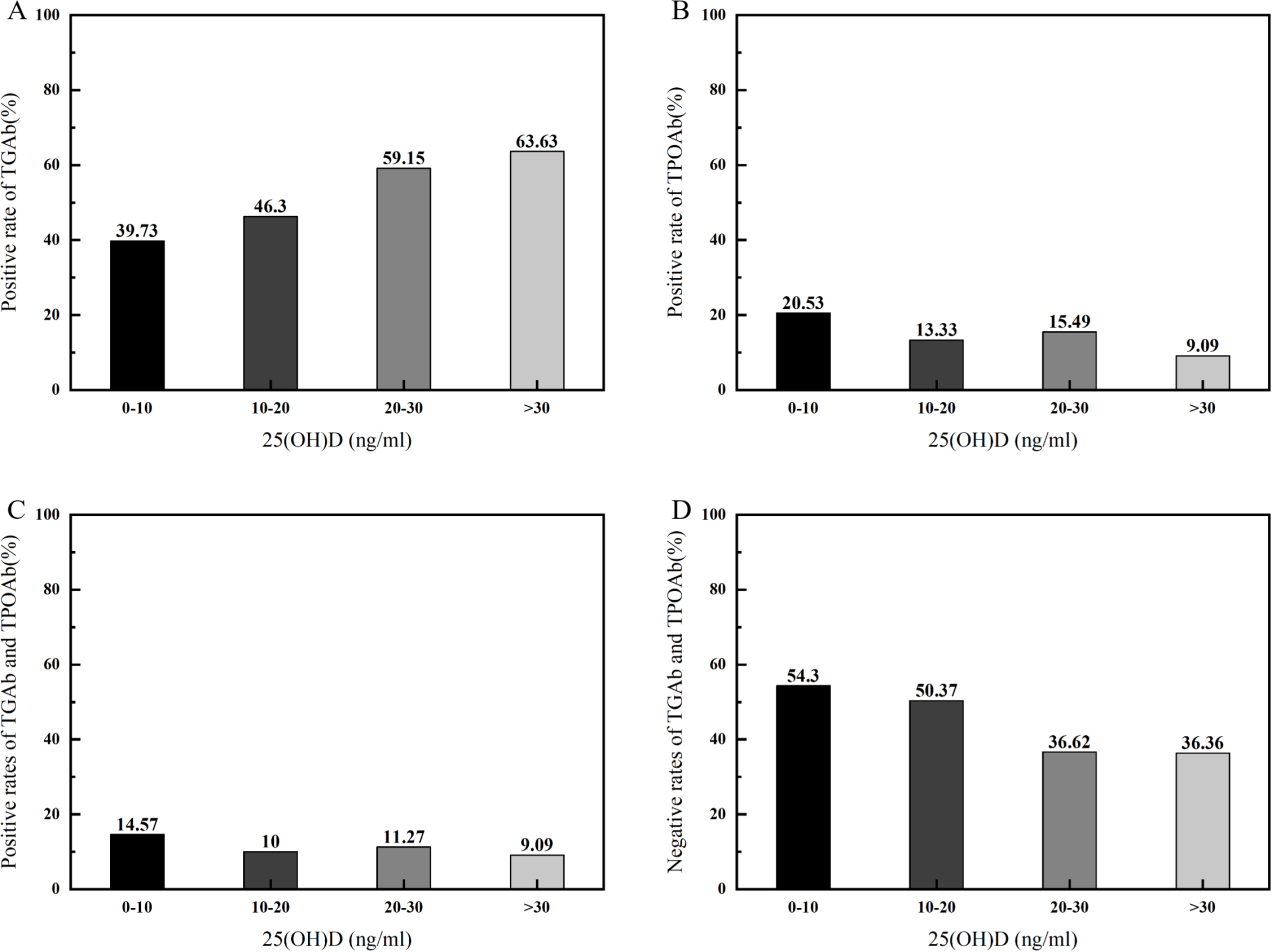
Antibody Positivity Rates according to 25(OH)D Levels. Prevalence of thyroid autoantibody positivity across four 25(OH)D strata: severe deficiency (<10 ng/mL), moderate deficiency (10–20 ng/mL), insufficiency (20–30 ng/mL), and sufficiency (≥30 ng/mL). **(A)** Positive rate of TGAb ; **(B)** Positive rate of TPOAb ; **(C)** Positive rates of TGAb and TPOAb; **(D)** Negative rates of TGAb and TPOAb.

Analysis of antibody positivity rates across 25(OH)D categories (0–10, 10–20, 20–30, ≥30 ng/mL) in **Table 4** revealed that TGAb positivity varied significantly according to 25(OH)D levels (P = 0.033), suggesting that 25(OH)D status may influence TGAb expression. In contrast, TPOAb positivity did not exhibit a significant association with 25(OH)D levels (P = 0.258), and dual antibody positivity (TGAb & TPOAb) was also not significantly associated with 25(OH)D (P = 0.618). Detailed antibody positivity rates across 25(OH)D categories are illustrated in **Figure 2**. **Figure 2a** shows that the TGAb positivity rate progressively increases across 25(OH)D categories (0–10, 10–20, 20–30, ≥30 ng/mL). This trend was not observed for other antibodies.

### 3.4 The association between 25(OH)D levels and thyroid autoantibodies

Based on **Tables 3**, **4**, the relationship between 25(OH)D levels and thyroid antibody status demonstrates statistically significant associations in specific subgroups. In **Table 3**, patients with positive TGAb exhibited significantly higher 25(OH)D levels than TGAb-negative patients (13.45 vs. 12.00 ng/mL, P = 0.008). Conversely, TPOAb-positive patients demonstrated lower 25(OH)D levels than TPOAb-negative patients (11.90 vs. 12.85 ng/mL, P < 0.001).

Analysis of antibody positivity rates across 25(OH)D categories (0–10, 10–20, 20–30, ≥30 ng/mL) in **Table 4** revealed that TGAb positivity varied significantly according to 25(OH)D levels (P = 0.033), suggesting that 25(OH)D status may influence TGAb expression. In contrast, TPOAb positivity did not exhibit a significant association with 25(OH)D levels (P = 0.258), and dual antibody positivity (TGAb & TPOAb) was also not significantly associated with 25(OH)D (P = 0.618). Detailed antibody positivity rates across 25(OH)D categories are illustrated in **Figure 2**. **Figure 2a** shows that the TGAb positivity rate progressively increases across 25(OH)D categories (0–10, 10–20, 20–30, ≥30 ng/mL). This trend was not observed for other antibodies.

### 3.5 Influence of TPOAb and TGAb positivity on 25(OH)D Status

In this study, participants were stratified into four groups according to TPOAb and TGAb status: TPOAb(+) TGAb(+), TPOAb(−) TGAb(+), TPOAb(+) TGAb(−), and TPOAb(−) TGAb(−) (**Table 5**). Non-parametric analysis using the Kruskal–Wallis test revealed a significant overall difference in 25(OH)D levels among the four groups (p = 0.002). Subsequent pairwise comparisons using the Mann-Whitney U test showed a statistically significant difference between the TPOAb(−) TGAb(+) and TPOAb(−) TGAb(−) groups (P < 0.001), while no significant differences were detected between TPOAb(+) TGAb(+) and TPOAb(+) TGAb(−) (P = 0.539), or between TPOAb(+) TGAb(−) and TPOAb(−) TGAb(−) (P = 0.604).

**Table 5:** Results of non-parametric statistical analysis of 25(OH)D levels stratified by TPOAb and TGAb status.

Group comparison	P value
TPOAb (+)/TGAb (+) vs TPOAb (-)/TGAb (-)	0.539
TPOAb (-)/TGAb (+) vs TPOAb (-)/TGAb (-)	<0.001*
TPOAb (+)/TGAb (-) vs TPOAb (-)/TGAb (-)	0.604
Combined comparison among all groups	0.002*

PS, ‘+’ indicates positive, ‘-’ indicates negative. The Kruskal-Wallis test was employed for comparisons among four groups, while the Mann-Whitney U test was used for between-group analysis of two groups. *indicates a statistically significant difference (P < 0.05).

**Figure 3:**
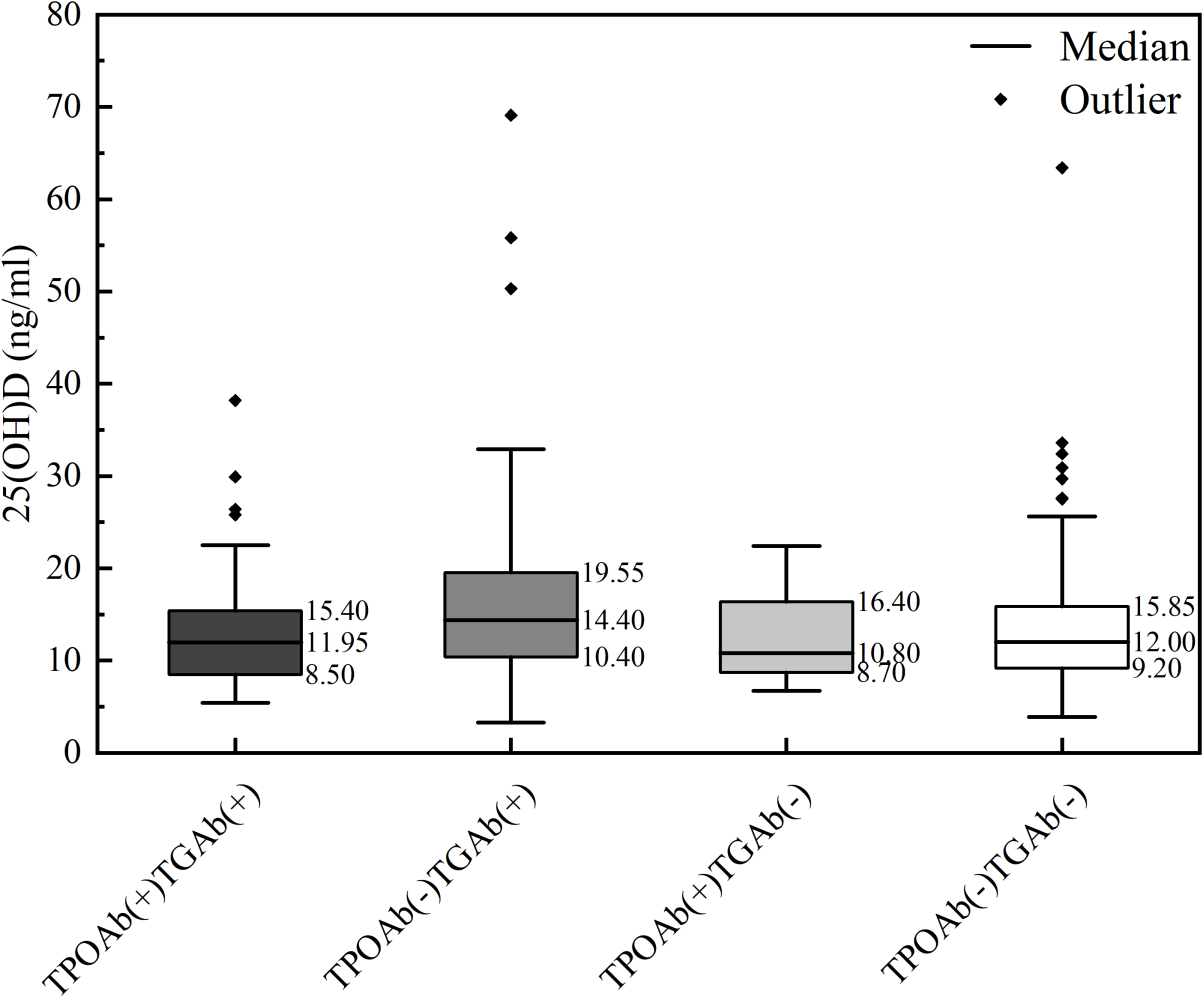
Distribution of serum 25(OH)D_3_ levels across thyroid autoantibody subgroups. Box plots depicting median 25(OH)D_3_ concentrations in four antibody-defined groups: TPOAb(+)TGAb(+), TPOAb(−)TGAb(+), TPOAb(+)TGAb(−), and TPOAb(−)TGAb(−). The TPOAb(−)TGAb(+) group showed the highest median level (14.40 ng/mL), while TPOAb(+) groups exhibited lower levels (11.95 and 10.80 ng/mL). Overall difference among groups was significant (Kruskal-Wallis, P = 0.002), with pairwise comparison revealing a significant difference between TPOAb(−)TGAb(+) and TPOAb(−)TGAb(−) groups (P<0.001). Outliers are shown as individual points.

Box plot analysis (**Figure 3**) depicts the distribution of 25(OH)D levels across the four antibody-defined groups. The TPOAb(−) TGAb(+) group exhibited the highest median level (14.40 ng/mL), followed by the TPOAb(−) TGAb(−) group (12.00 ng/mL). The TPOAb(+) TGAb(+) and TPOAb(+) TGAb(−) groups displayed lower medians (11.95 ng/mL and 10.80 ng/mL, respectively). Notably, several outliers were identified in the TPOAb(+) TGAb(+) and TPOAb(−) TGAb(+) groups. These findings suggest that individuals with TGAb positivity, particularly those lacking TPOAb, tend to exhibit higher 25(OH)D levels, whereas TPOAb positivity may be associated with lower levels.

### 3.6 Analysis of multiple linear regression model fit

To further investigate factors independently associated with serum 25(OH)D levels in patients with T2DM, variables showing statistical significance in the univariate analysis (T3, fT3, PTH, hsCRP, Ca, TPOAb, and TGAb) were entered into a multiple linear regression model, with serum 25(OH)D concentration as the dependent variable.

**Figure 4:**
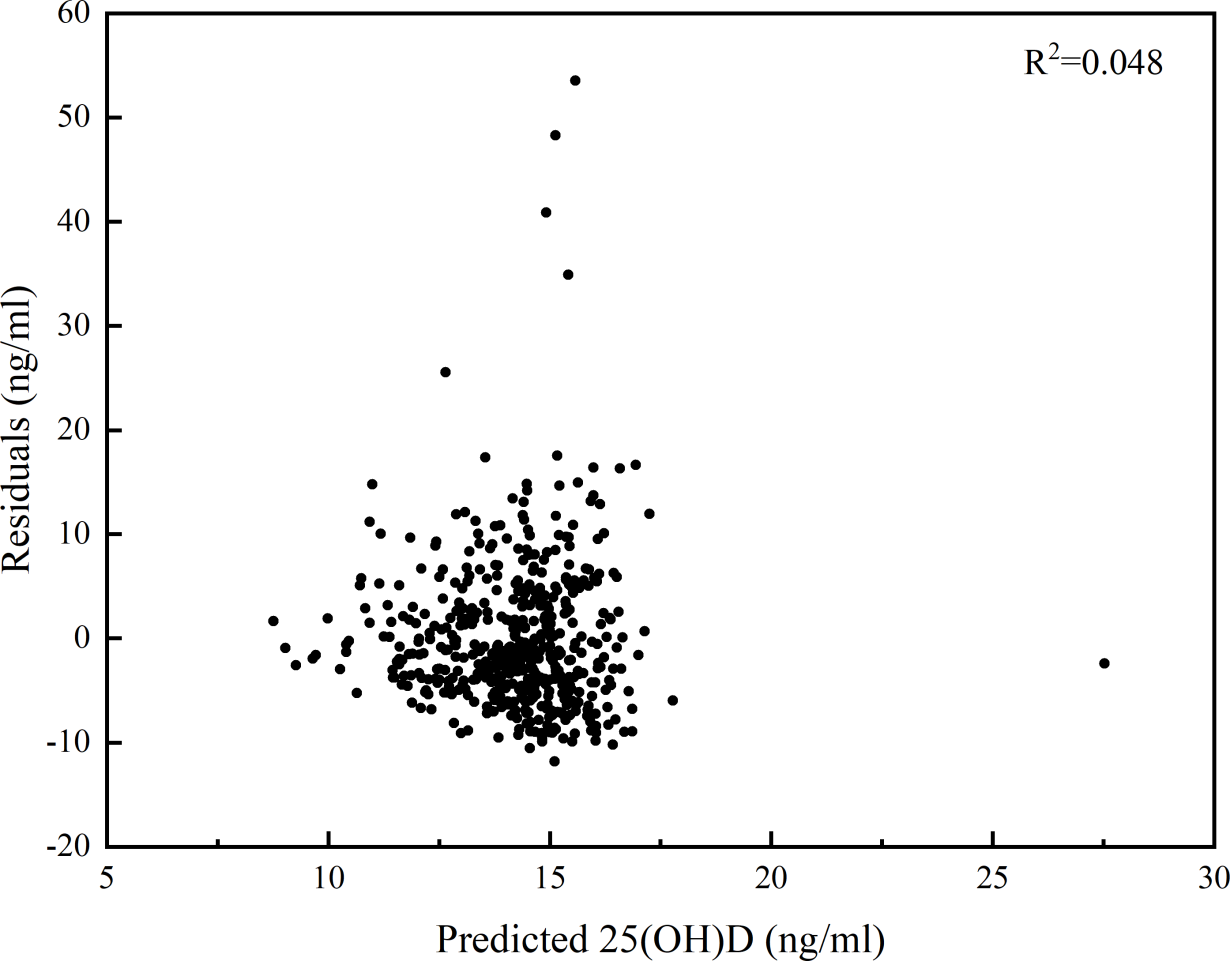
Residual Analysis of Multiple Linear Regression Model. Residual plots from the regression model predicting 25(OH)D levels using T3, fT3, PTH, hsCRP, calcium, TPOAb, and TGAb. The plot of residuals versus predicted values shows a symmetric distribution centered around zero, with no systematic patterns, indicating absence of model bias. Residual dispersion remains constant across predicted values, demonstrating homoscedasticity. A few isolated outliers do not compromise model robustness. The model exhibits satisfactory fit and good predictive capability.

Following model construction, we evaluated its goodness-of-fit through residual analysis (**Figure 4**). Residual plots indicated that the model achieved a satisfactory overall fit, as evidenced by the following features: a centered and symmetric residual distribution, where the vast majority of residuals clustered tightly around zero, suggesting approximate normality (R^2^ = 0.048); and the absence of systematic bias or heteroscedasticity, as no discernible patterns were observed in the residual distribution and the dispersion did not increase with predicted values. These findings indicate the absence of global model bias or heteroscedasticity. Although a small number of observations (isolated outliers) exhibited larger prediction errors, they did not substantially affect the model’s overall robustness. The results of the multiple linear regression analysis are presented in **Table 6**. Among all included variables, only PTH was significantly associated with the outcome (β = -0.049, P = 0.001), indicating a negative independent relationship. Specifically, higher PTH levels were associated with lower values of the dependent variable. Other variables, including T3, fT3, hsCRP, Ca, TPOAb, and TGAb, showed no statistically significant associations (P > 0.001).

**Table 6:** Multiple linear regression results.

Clinical laboratory indicators	β	SE	t	P value	CI_Lower	CI_Upper	VIF
T3	0.495	1.095	0.452	0.652	-1.656	2.646	3.37
fT3	0.320	0.436	0.735	0.463	-0.536	1.177	3.35
PTH	-0.049	0.015	-3.390	0.001	-0.078	-0.021	1.01
hsCRP	-0.008	0.062	-0.126	0.900	-0.130	0.114	1.01
Ca	-0.061	0.072	-0.844	0.399	-0.203	0.081	1.00
TPOAb	-0.002	0.001	-1.333	0.183	-0.004	0.001	1.45
TGAb	-0.001	0.004	-0.259	0.796	-0.010	0.008	1.43

SE, Standard Error; VIF, Variance inflation factor.

No significant multicollinearity was observed among the included variables, as all variance inflation factor (VIF) values were below 5.

## 4 Discussion

In this cohort of 503 patients with T2DM and concurrent HT, we observed a high prevalence of 25(OH)D deficiency (83.7%), consistent with previous reports (18–21). Our findings extend previous observations by demonstrating that vitamin D’s relationships with the calcium-PTH axis, systemic inflammation (hsCRP), and peripheral thyroid hormone metabolism (T3/fT3) are more nuanced than simple linear correlations, while confirming the absence of significant associations with glycemic control, body composition, or bone turnover markers.

Consistent with established physiological mechanisms (22, 23), PTH levels progressively declined and serum calcium increased with higher 25(OH)D concentrations, supporting the role of 25(OH)D in calcium homeostasis and the negative feedback regulation of PTH. The absence of a significant association with bone formation markers (osteocalcin, phosphorus) contrasts with studies in populations with diabetic kidney disease, where such links are pronounced (23, 24). This discrepancy may be attributed to the exclusion of patients with significant renal impairment in our study, highlighting the influence of renal function on 25(OH)D’s skeletal effects.

Regarding inflammation, we observed a non-monotonic association between 25(OH)D and hsCRP, with the highest inflammatory burden in the moderate deficiency group (10–20 ng/mL). This pattern supports a potential threshold effect of vitamin D on systemic inflammation. Consistent with previous evidence linking 25(OH)D status to inflammatory and metabolic regulation (25). Similarly, Kositsawat et al. further reported that low 25(OH)D and elevated CRP jointly increase diabetes risk (26). Furthermore, the lack of association with HbA1c and BMI in our study is corroborated by large trials, which found no glycemic benefit from 25(OH)D supplementation in prediabetes (27). This underscores that 25(OH)D’s primary clinical impact in T2DM may not be on core metabolic parameters.

A particularly noteworthy finding was the selective reduction of T3 and fT3 in patients with severe 25(OH)D deficiency (<10 ng/mL), while T4, fT4, and TSH remained stable. This pattern suggests that vitamin D may influence peripheral thyroid hormone metabolism rather than central synthesis. Mechanistically, 25(OH)D response elements have been identified in the promoters of deiodinase enzymes (DIO1, DIO2), which catalyze T4-to-T3 conversion (28). The observation that T3/fT3 levels peaked in the insufficiency range (20–30 ng/mL; T3: 1.75 nmol/L, fT3: 5.02 pg/mL) and declined in sufficiency further suggests a potential “optimal window” for 25(OH)D in supporting peripheral thyroid hormone activation. One plausible explanation for this inverted U-shaped pattern is that moderate 25(OH)D levels optimize deiodinase gene transcription via vitamin D response elements, whereas severe deficiency fails to provide sufficient ligand for VDR activation, and very high levels may over-suppress the hypothalamic-pituitary-thyroid axis or compete with other nuclear receptors.

The relationship between 25(OH)D and thyroid autoantibodies revealed a nuanced dissociation between TPOAb and TGAb. TGAb-positive patients, particularly those lacking TPOAb, exhibited higher median 25(OH)D levels (14.40 ng/mL), whereas TPOAb positivity was associated with lower levels (11.90 vs. 12.85 ng/mL, P<0.001). This contrasts with some previous studies reporting an inverse relationship between 25(OH)D status and thyroid autoimmunity (29–34), suggesting that coexisting T2DM or other metabolic factors may modulate this association. One possible explanation involves differential immune pathway regulation: TPOAb production is closely linked to Th1-mediated cytotoxic responses, while TGAb can arise from Th2-dominant environments and may even represent a regulatory phenomenon (35). 25(OH)D is known to suppress Th1 responses while promoting regulatory T-cell differentiation (36). In the context of T2DM—characterized by chronic low-grade inflammation and Th1 skewing—25(OH)D’s immunomodulatory effects may differentially impact these antibody profiles. These findings underscore the importance of considering both antibody profiles and metabolic context when evaluating vitamin D status in autoimmune thyroid disease. Clinically, isolated TGAb positivity is often considered less pathogenic than TPOAb positivity, and it can occur in healthy individuals without thyroid dysfunction. Therefore, the higher 25(OH)D level in TGAb-positive, TPOAb-negative individuals might reflect a state of immune regulation rather than ongoing thyroid destruction.

A key strength of this study is the development of an exploratory multivariable model integrating T3, fT3, PTH, hsCRP, calcium, TPOAb, and TGAb to identify T2DM patients at risk for significant vitamin D deficiency—a prevalent subgroup comprising over 80% of this population (37–39). This model focuses on novel, non-traditional markers, moving beyond conventional parameters such as HbA1c and BMI, and may facilitate targeted screening in clinical settings. Our findings should be interpreted alongside recent study. Wu et al. (40) reported a positive linear association between 25(OH)D and FT3 in 1,805 T2DM patients, but did not examine non-linearity. Our results extend these reports by identifying a non-linear “optimal window” (20–30 ng/mL) for T3/fT3, and by demonstrating that TGAb and TPOAb follow divergent patterns relative to vitamin D status. These nuances would have been missed by linear models alone, underscoring the value of stratified analyses.

Several limitations should be acknowledged. The cross-sectional design precludes causal inference regarding the directionality of the observed associations—for instance, whether 25(OH)D deficiency contributes to reduced T3 levels, or whether shared factors (such as illness-related inflammation) independently lower both parameters. The study population was restricted to T2DM patients with HT, potentially limiting generalizability to other populations. In addition, seasonal variation in 25(OH)D levels was not accounted for, as samples were collected throughout the year, which may have introduced variability in measured concentrations. Future studies should consider seasonal adjustment or standardized sampling periods. Despite these limitations, the study provides valuable insights into the interplay between 25(OH)D, calcium-PTH metabolism, systemic inflammation, and thyroid function in a clinically relevant population.

Clinically, our findings suggest that maintaining 25(OH)D above 20 ng/mL in T2DM patients with HT may support both calcium-PTH balance and inflammatory control. The observed decline in T3/fT3 at levels <10 ng/mL further implies that preventing severe deficiency could have implications for thyroid hormone metabolism. Moreover, the differential antibody profiles observed suggest that vitamin D’s immunomodulatory effects may vary based on the specific autoimmune phenotype, potentially informing personalized supplementation strategies, although interventional studies are necessary to validate this approach.

Future research should prioritize longitudinal and interventional studies to clarify causal relationships between 25(OH)D and thyroid hormone metabolism, inflammatory status, and antibody expression. Mechanistic studies examining vitamin D’s effects on deiodinase activity and Th1/Th2 polarization in the context of T2DM would be particularly valuable. Such investigations could elucidate the potential benefits of correcting 25(OH)D deficiency in patients with T2DM and HT and may guide personalized strategies for managing thyroid autoimmunity and metabolic health.

## 5 Conclusions

This study addresses three key gaps in the literature on vitamin D in T2DM. First, we demonstrate a non-linear, inverted U-shaped relationship between 25(OH)D and T3/fT3 levels, peaking at 20–30 ng/mL – suggesting an optimal window for peripheral thyroid hormone conversion. Second, we reveal a dissociation in thyroid autoimmunity: TGAb positivity is associated with higher, and TPOAb positivity with lower 25(OH)D levels. Third, we provide a comprehensive evaluation showing that vitamin D deficiency (83.7% prevalence) significantly impacts the calcium-PTH axis (PTH decreasing, calcium increasing) and systemic inflammation (hsCRP peaking at moderate deficiency), while no associations are found with glycemic control, BMI, T4/fT4/TSH, or OC. These findings highlight the importance of assessing and addressing vitamin D deficiency in T2DM patients.

## Data Availability

The raw data supporting the conclusions of this article are not publicly available due to the restrictions imposed by the Ethics Committee of the Fourth People’s Hospital of Shenyang on patient privacy. Anonymized data may be made available by the corresponding author upon reasonable request, subject to ethical approval.
