# Supplementary material for: Association of 25(OH)D status with calcium metabolism, inflammation, and thyroid autoimmunity in patients with type 2 diabetes mellitus
Source: Front Endocrinol (Lausanne). 2026 Jun 4;17:1867755. doi: 10.3389/fendo.2026.1867755 (PMC13275219; doi:10.3389/fendo.2026.1867755)
Supplement: Supplementary file 1 [file Table1.docx]

**Table 1.** Normality test of continuous variables.

| Clinical Laboratory Indicators | Skewness | Kurtosis | Shapiro-Wilk W | P value | Distribution |
| --- | --- | --- | --- | --- | --- |
| Age | -0.707 | 0.383 | 0.964 | <0.001* | Non-normal |
| Diabetic duration | 0.827 | 0.601 | 0.948 | <0.001* | Non-normal |
| BMI | 1.591 | 10.493 | 0.919 | <0.001* | Non-normal |
| T3 | 11.611 | 200.693 | 0.473 | <0.001* | Non-normal |
| T4 | -0.004 | 0.417 | 0.990 | 0.002* | Non-normal |
| fT3 | 13.111 | 226.150 | 0.360 | <0.001* | Non-normal |
| fT4 | 15.913 | 310.326 | 0.301 | <0.001* | Non-normal |
| sTSH | 14.759 | 245.691 | 0.183 | <0.001* | Non-normal |
| hsCRP | 6.845 | 59.348 | 0.366 | <0.001* | Non-normal |
| PTH | 1.225 | 2.277 | 0.925 | <0.001* | Non-normal |
| OC | 2.619 | 18.094 | 0.850 | <0.001* | Non-normal |
| HbA1c | 0.740 | 0.060 | 0.954 | <0.001* | Non-normal |
| Ca | 21.972 | 483.177 | 0.030 | <0.001* | Non-normal |
| P | 0.523 | 1.793 | 0.982 | <0.001* | Non-normal |

The Shapiro–Wilk test was used for analysis.
